# Modified Silica Nanoparticles from Rice Husk Supported on Polylactic Acid as Adsorptive Membranes for Dye Removal

**DOI:** 10.3390/ma16062429

**Published:** 2023-03-18

**Authors:** João Otávio Donizette Malafatti, Francine Aline Tavares, Tainara Ramos Neves, Bruno Cano Mascarenhas, Simone Quaranta, Elaine Cristina Paris

**Affiliations:** 1 Nanotechnology National Laboratory for Agriculture (LNNA), Embrapa Instrumentação, São Carlos 13560-970, Brazil; 2 Department of Chemistry, Federal University of São Carlos, São Carlos 13565-905, Brazil; 3 Institute for the Study of Nanostructured Materials, Italian National Research Council (ISMN–CNR), 00010 Rome, Italy

**Keywords:** silica nanoparticles, rice husk, PLA, electrospinning, nanocomposite, adsorption

## Abstract

Industrial effluents and wastewater treatment have been a mainstay of environmental preservation and remediation for the last decade. Silica nanoparticles (SiO_2_) obtained from rice husk (RH) are an alternative to producing low-cost adsorbent and agriculture waste recovery. One adsorption challenge is facilitating the adsorbate separation and reuse cycle from aqueous medium. Thus, the present work employs SiO_2_ supported on polylactic acid (PLA) nanofibers obtained by the electrospinning method for Rhodamine B (RhB) dye adsorption. The silica surface was modified with trimethylsilyl chloride (TMCS) to increase affinity towards organic compounds. As a result, the silanized surface of the silica from rice husk (RHSil) promoted an increase in dye adsorption attributed to the hydrophobic properties. The PLA fibers containing 40% SiO_2_ (w w^−1^) showed about 85–95% capacity adsorption. The pseudo-first-order kinetic model was demonstrated to be the best model for PLA:SiO_2_ RHSil nanocomposites, exhibiting a 1.2956 mg g^−1^ adsorption capacity and 0.01404 min^−1^ kinetic constant (k_1_) value. In the reuse assay, PLA:SiO_2_ membranes preserved their adsorption activity after three consecutive adsorption cycles, with a value superior to 60%. Therefore, PLA:SiO_2_ nanocomposites from agricultural waste are an alternative to “low-cost/low-end” treatments and can be used in traditional treatment systems to improve dye removal from contaminated waters.

## 1. Introduction

Environmental remediation processes have been applied in waste treatment to minimize the ecological impacts occasioned by human actions [1,2]. The adsorption of pollutants with low-cost adsorbents is controlled mainly by physical interactions, making this process more attractive for wastewater treatment since this characteristic allows the adsorbent to be reused in many cycles [3,4]. Among the desirable features of adsorbents are the high specific surface area (SSA), easy regeneration, and low-cost production [5]. In this context, activated carbon is the most used material due to its efficient adsorption capacity [6,7,8]. On the other hand, nanomaterials are a promising alternative due to the improvement of properties through surface and structural modifications [9,10,11,12]. Therefore, many researchers have studied alternative adsorbents that can efficiently substitute for activated carbon, including agricultural residues (sugar cane bagasse, coconut husk, corn cob) [13], zeolites [14,15], and silica [16,17,18], among others [19,20].

Silica (SiO_2_) has a tetrahedral structure in which the silicon atom occupies the central position coordinated with four oxygen atoms [21]. As a result, crystalline forms, such as quartz, tridymite, and cristobalite, could be formed. On the other hand, the random arrangement of tetrahedra characterizes silica in the amorphous (non-crystalline) form [22]. SiO_2_ nanoparticles are distinct compounds with efficient surface chemical reactivity, thermal stability, a high surface area, and a porous structure [23,24]. Therefore, silica nanostructures have been widely used for applications in catalysis, thermal insulators, sensors, and adsorption [25]. Although SiO_2_ nanoparticles can be synthesized efficiently by the sol–gel process [26], rice husk extraction is an alternative to obtaining high-purity silica from an agribusiness residue. The main constituents of rice husks are lignin, cellulose, and hemicellulose, accounting for about 80% of the total mass, and 17% of the mineral ash [27,28,29]. Thus, the rice husk is a natural residue that, with the appropriate extraction method, can produce high-purity silica with a high SSA from 250 to 900 m^2^ g^−1^ by decomposition of organic compounds [30,31].

The efficiency of removing organic pollutants from aqueous solutions can be increased by modifying the adsorbent surface, expanding the material hydrophobicity, and improving the interaction with the pollutant molecules [4,32]. The high chemical reactivity of silica originates from the efficient surface covering by silane groups (Si-O-H) from R_n_SiX_(4−n)_ compounds, where R is the organic part that can be an alkyl or organofunctional group and X is an alkoxy group (ethoxy or methoxy) or a halide [33,34]. Roe and Zang [35] studied the surface hydrophobization of silica nanoparticles for different silane sources. These authors obtained surfaces with maximum hydrophobicity using the combination of n-octyltrimethoxysilane (OTMS) and bis(triethoxysil)ethane (BTEOSE), producing a contact angle of 139.1°. Velmurugan et al. [36] obtained functionalized silica modified with amino groups from 3-aminopropyltriethoxysilane and evaluated the efficiency against the anionic dye Congo red adsorption. The pristine silica showed around 40% of dye removal and 98% after the surface modification. The authors attributed the high adsorption efficiency to the attractive forces between the adsorbent amine group and the dye molecules. In this context, silica with a hydrophobic nature and highly efficient adsorption of organic contaminants can be produced from functionalization reactions with suitable silane reagents. However, removing particulate solids from aqueous solutions, especially in nanometer scales, induce a challenge due to the complex, large-scale application of these materials [37].

The use of supports in heterogeneous adsorption methods promotes an adsorbent-accessible recovery and simple separation from the aqueous medium [38,39,40,41]. Additionally, nanocomposites combine individual characteristics to increase desirable properties [42,43,44,45]. Fibrous membranes are one alternative to immobilizing the nanoparticles due to the porosity and diameter characteristics improving the surface area [46,47]. Specifically for adsorption process applications, using nanoparticles immobilized on fibers aims to facilitate the adsorbent material’s removal from the aqueous medium and promote its reuse for many cycles [48]. One technique to produce fibrous membranes is electrospinning, a simple and practical way to introduce nanoparticles into a polymer matrix [32,37,49]. Xu et al. [50] obtained polyacrylic acid (PAA) fibers with SiO_2_ functionalized with mercapto silane groups with a maximum indigo carmine dye adsorption of 523 mg g^−1^. In further work [47], this group used the PAA:SiO_2_ membrane to adsorb malachite green dye. In the results, the membranes demonstrate stability after multiple cycles without significant loss of their adsorption capacity. Among the different polymers, polylactic acid (PLA) has gained special attention attributed to its non-toxicity, biodegradability, and flexibility [51,52]. Therefore, PLA has been used in applications such as biomedical [53], active packaging [54], and remediation supports [14,55]. From this perspective, a system based on PLA:SiO_2_ fibers shows desirable characteristics for a membrane to be applied in adsorption processes.

The great potential stemming from joining SiO_2_ nanoparticles has been investigated recently. For instance, PLA/silica composites have been successfully applied to packaging [56] and oil removal [57]. Nevertheless, studies concerning PLA:SiO_2_ membranes for water remediation (i.e., dye elimination) are scarce. Furthermore, research has focused on high-cost, synthetic silica-based solutions for removing water contaminants. On the other hand, plenty of waste sources are available to synthesize silica nanoparticles. Therefore, the present study developed adsorbing membranes predicated on low-cost silica nanoparticles incorporated into silica particles. Specifically, mesoporous silica was attained from rice husk agri-waste by simple acid leaching and thermal annealing. Then, this material was supported on PLA fibers measuring up to both commercial and sol-gel synthesized silica nanoparticles for Rhodamine B removal. In addition, the SiO_2_ external surface was modified with the trimethylsilyl chloride silane (TMSCl) to promote interaction with Rhodamine B (RhB). Thus, the proposed nanocomposite material can be regarded as a “low-end” platform for the adsorption of pollutants from aqueous solutions. In fact, rice-husk-derived PLA:SiO_2_ membranes are suitable for recovery and multiple reuses.

## 2. Materials and Methods

### 2.1. Materials

Silica commercial (AC), tetraethyl orthosilicate (TEOS), trimethylsilyl chloride (TMSCl), ammonium hydroxide (NH_4_OH), chloroform, and RhB were purchased from Sigma-Aldrich^®^ (St. Louis, MO, USA). Dimethylsulfoxide (DMSO) was purchased from Labsynth^®^ (Diadema, Brazil). Toluene was purchased from Dinâmica^®^ (Indaiatuba, Brazil). All chemicals were used as received without further purification.

### 2.2. SiO_2_ from Rice Husk Synthesis (RH)

Rice husk residues (RHs) were used to prepare the RH SiO_2_ sample, as reported in the literature [31,58,59]. First, pristine RHs were washed with deionized water and dried overnight in an oven at 100 °C. Then, in sequence, 10 g of dried RHs were soaked into a 3M HCl solution (200 mL) and maintained under stirring and reflux at 80 °C. After 3 h, the RHs were removed from the leaching solution, washed to neutral pH with distilled water, and dried in an oven at 80 °C. Finally, the material was annealed in a muffle furnace at 600 °C for 3 h. The characterization results and RhB adsorption performances of the prepared silica nanoparticles were compared and contrasted to commercially available nanostructured Sigma-Aldrich silica (AC) and synthetic silica obtained by the sol–gel method (SG).

### 2.3. SiO_2_ by Sol–Gel Synthesis (SG)

SG SiO_2_ nanoparticles were synthesized by the sol–gel method according to the procedure reported by Stöber [60]. Briefly, 8 mL of NH_4_OH and 7.5 mL of tetraethyl orthosilicate were added to an aqueous ethanol solution (1:1.5 V V^−1^) in a round bottom flask. The mixture was maintained under vigorous stirring for 1 h at room temperature. After that, solvents were evaporated to attain a solid residue. Next, this material was thermally treated for 3 h at 350 °C and subsequently at 400 °C for 2 h.

### 2.4. SiO_2_ Surface Modification with Trimethylsilyl Chloride (TMSCl)

The TMSCl was used to functionalize SiO_2_ surface nanoparticles in a procedure adapted from Kulkarni et al. [33,61]. At room temperature, 1.0 g of dry SiO_2_ was dispersed in 15 mL of toluene by constant stirring for 1 h. Then, 2 mL of TMSCl was added, and the mixture was kept under reflux for 48 h. The obtained material was washed with toluene and dried in an oven at 80 °C. Silanized samples were labeled XSil, where X is AC, SG, or RH.

### 2.5. PLA:SiO_2_ Silanized Fibers

Silanized SiO_2_ nanoparticles (Sil) were immobilized on PLA fibers by electrospinning. According to the methodology described above, PLA was initially solubilized in a mixture of chloroform and dimethylsulfoxide in a 3:1 (V V^−1^) ratio. After complete PLA solubilization, modified SiO_2_ was added, and the solution was continuously stirred at room temperature for 2 h. After homogenizing, the mixture was transferred to a glass syringe to electrospin the PLA:SiO_2_ fibers. A 40 wt% silica load (concerning PLA mass) was chosen for the composite fabrication. Electrospinning parameters were set as follows: applied voltage at 20 kV, needle tip-to-collector distance equal to 5 cm, and flow rate at 3 mL h^−1^.

### 2.6. Characterizations

Silica particles and PLA:SiO_2_ Sil composite fibers were fully characterized (i.e., by structure, composition, morphology). Specifically, the crystallographic phase composition of SiO_2_ particles was investigated by X-ray diffraction (XRD). A Lab-XRD-6000 Shimadzu^®^ (Kyoto, Japan) diffractometer equipped with a Cu-Kα radiation source (λ = 1.5406 Å), 2θ angular range from 5° to 80°, and a scanning step of 1° min^−1^ was used. Chemical and structural features were obtained by ^29^Si solid-state nuclear magnetic resonance (NMR) spectroscopy performed with a Bruker (Billerica, MA, USA) Avance III HD 400 MHz spectrometer operating at 10 kHz. Thermogravimetric analysis (TGA) was exploited to verify the complete removal of organic residues in the RH SiO_2_ sample and quantify the composite fibers’ silica amount. TGA was carried out using a Netzsch (Selb, Germany) model STA 409^®^ equipment in an alumina pan, from room temperature to 900 °C under nitrogen flux and with a 10 °C min^−1^ scan rate. The morphology of SiO_2_ particles and fibers was investigated by scanning electron microscopy (SEM) using a JEOL (Tokyo, Japan) JSM-6510 field emission electron microscope equipped with an EDS (electron dispersion spectroscopy) detector for compositional analysis. Finally, particle size was measured by transmission electron microscopy (TEM). Therefore, the samples were dispersed in acetone through ultrasonication and analyzed with a FEI^®^ (Hillsboro, OR, USA) TECNAI G2 F20 microscope. The specific surface area (SAA), micropore area (A_Pore_), external area (A_Ext_), and pore diameter of the powder samples were determined by nitrogen adsorption/desorption through the Brunauer–Emmett–Teller (BET) method. Zeta potential and contact angle measurements were performed on pristine and TMSCl-functionalized SiO_2_ particles (SG and RH) using a Malvern Instruments^®^ (Malvern, UK), Zetasizer Nano ZS90 and an Optical contact angle and surface tension meter model CAM100 by KSV, respectively.

### 2.7. Rhodamine B Adsorption Assays

#### 2.7.1. SiO_2_ Nanoparticles

SiO_2_-free unmodified and TMSCl-modified particles were evaluated to verify the efficiency of the different samples: SiO_2_ extracted from rice husk, sol–gel synthesis, and commercial SiO_2_. Thus, 40 mg of each adsorbent was added to an aqueous solution (40 mL) containing 5 mg L^−1^ of RhB dye and continuously stirred for 24 h at a neutral pH. At the end of the tests, powder adsorbents were removed by centrifugation. Finally, the residual RhB concentration was evaluated by UV-Vis absorption spectroscopy (with a Shimadzu model UV-1601PC).

#### 2.7.2. PLA:SiO_2_ Sil Membranes

The RhB dye adsorption was initially carried out with different adsorbent concentrations (1, 2, and 4 g L^−1^) to determine the ideal amount to achieve high adsorption efficiencies. First, the test was performed in RhB solution (5 mg L^−1^, 20 mL) under continuous stirring for 24 h at 25 °C. Then, the adsorbents were removed manually, and the RhB residual solution concentration was determined by UV-Vis spectroscopy.

Subsequently, the time required to reach equilibrium to remove the maximum percentage of the dye was evaluated. A kinetic study was performed for 2 and 4 g L^−1^ of PLA:SiO_2_ SGSil and PLA:SiO_2_ RHSil, respectively. The dye removal was monitored at different time points (30, 60, 120, 180, 240, and 1440 min). The kinetic study for the RhB dye adsorption with the membranes was performed by applying the pseudo-first-order (Equation (1)) and pseudo-second-order (Equation (2)) models according to the following equations:

Pseudo-first order:

(1) $$\;\log\left(q_{e}-q_{t}\right)=\log\;q_{e}-\frac{k_{1}}{2.303}\;t\;$$

Pseudo-second order:

(2) $$\frac{t}{q_{t}}=\frac{1}{k_{2}q_{e}^{2}}+\frac{1}{q_{e}}\;t$$

where *q_e_* (mg g^−1^) is the RhB amount adsorbed at equilibrium, *q_t_* (mg g^−1^) is the amount adsorbed at time *t* (min), and *k*_1_ (min^−1^) and *k*_2_ (g mg^−1^ min^−1^) are the rate constants for the pseudo-first-order and pseudo-second-order reactions, respectively. According to the literature [62,63,64], these kinetic models are the ones most commonly applied for RhB adsorption systems, presenting the best fit for the experimental results.

A reuse assay was performed to verify the stability of the adsorbent membranes for RhB dye removal. The evaluation up to three reuse cycles was realized using the best response conditions for the adsorption process. In this stage, the reused fibers were subjected to the RhB adsorption/desorption process. After the recovery, the membranes were washed using ethanol and dried to undergo a new cycle. All the experiments were replicated thrice under agitation at 25 °C for 180 min.

## 3. Results and Discussion

**Figure 1:**
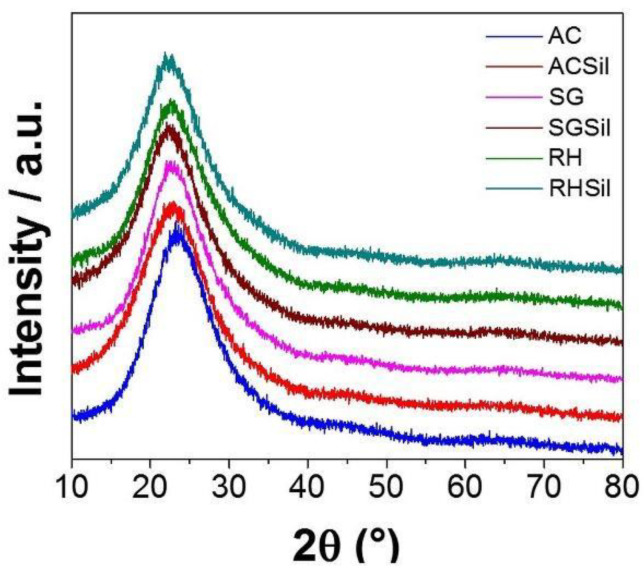
XRD diffractograms for SiO_2_ with unmodified surface and modified surface with TMSCl silane.

The XRD patterns concerning SG and RH are reported in Figure 1. The commercial silica (AC) diffractogram is also inserted for comparison in Figure 1. All samples presented a broad diffraction peak at 2θ = 22.5°, a distinctive feature of amorphous (i.e., poorly crystalline) silica [25]. There is significant evidence that the RH sample showed no crystalline reflections of impurities from the original raw material. The AC nanoparticles were found to be made out of pure, mainly amorphous, silica. Furthermore, TMSCl functionalization did not affect the materials’ crystal structure or composition (see Figure 1).

**Figure 2:**
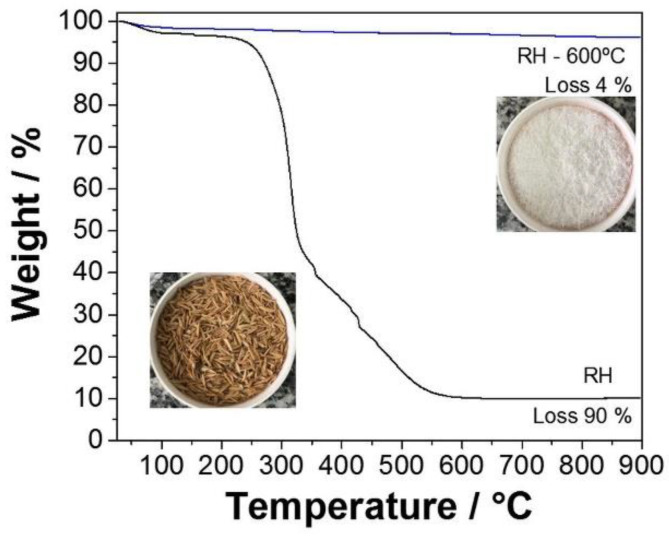
Thermogravimetric analysis of SiO_2_ before and after rice husk (RH) treatment at 600 °C.

Thermal analysis was performed on pristine and acid-treated rice husks (Figure 2) to confirm the RH purity. Moreover, TGA curves were also acquired for acid-treated rice husks before and after calcination at 600 °C.

Regardless of the specific treatment, the sample without heat treatment presented a mass loss of 2% at about 70 °C, corresponding to the release of physisorbed water. Additionally, a considerable mass loss of 6%, beginning at about 250 °C (inflection point at 330 °C), was attributed to the rapid cracking of cellulose, hemicellulose, and lignin [65]. An additional loss of 82% after 490 °C was attributed to the complete removal of cellulose, hemicellulose, and lignin degradation products [66]. As expected, the acid-treated rice husk’s total mass loss was more significant than the pristine material. Indeed, acid leaching could remove rice husk’s inorganic components (mainly potassium and calcium). In addition, the thermal treatment causes the crystallization of potassium and calcium oxides, acting as trapping centers for carbon residues [67]. Thus, HCl leaching can be considered instrumental in attaining high-purity SiO_2_. Moreover, the acid-treated silica calcined at 600 °C presented a negligible mass loss indicating the complete elimination of any organic impurities, as was suggested by the visual inspection of the sample (white color). This fact was supported by the XRD results (Figure 1).

**Figure 3:**
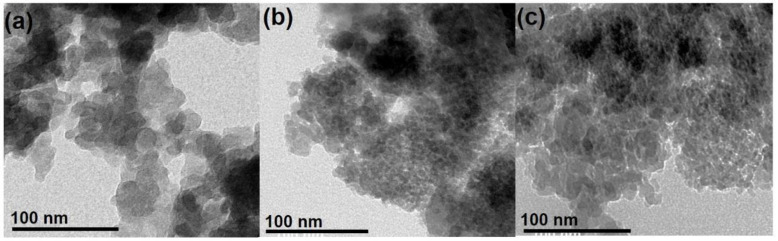
SEM images of SiO_2_ nanoparticles: (**a**) AC, (**b**) SG, and (**c**) RH.

TEM images of commercial and synthetic SiO_2_ are illustrated in Figure 3. The former comprises well-separated, quasi-spherical nanoparticles with an average diameter of around 15–16 nm. On the other hand, small particle sizes (5–10 nm) compared to AC were observed concerning SG and RH. As for SG, the formation of tiny particles was most likely favored by the large H_2_O/NH_3_ ratio used during the synthesis. In addition, water triggers TEOS hydrolysis, generating plenty of nuclei in the initial reaction stages. Conversely, the limited amount of ammonia keeps the nuclei from growing. Concerning RH, particle size is mainly determined by the annealing temperature. Usually, 600 °C is considered an efficient trade-off between high reactivity assured by small particle (i.e., large specific surface area) size and the complete removal of organic matter [30,31,58,66].

Specific surface area (SSA) data and pore size salient parameters for the silica samples (before and after TMSCl treatment) are reported in Table 1. In particular, the average particle size (estimated by TEM), micropore area (A_Pore_), external area (A_Ext_), total surface area (A_Pore_ + A_Ext_ = SSA_BET_), and pore diameter are reported.

SSA values partially reflected the particle size and the agglomeration degree of the samples. Therefore, RH had the lowest SSA_BET_ (246 m^2^ g^−1^) because of particle clustering brought about by the thermal treatment required to burn off the organics. On the other hand, SG SSA_BET_ (537 m^2^ g^−1^) benefited from the small particle size deriving from the excess water utilized in the material preparation. Despite larger particle sizes, AC SSA_BET_ (527 m^2^ g^−1^ ) proved to be similar to SG due to the low level of particle clustering. In addition, AC SSA_BET_ was almost equally shared between external and pore surface area.

On the contrary, the external surface area can be regarded as the only contribution to SSA_BET_ for RH and SG. The lack of porous structure for the two prepared samples is expected from the nature of the synthetic procedure. Indeed, the RH and SG preparation routes employed no templating agents (e.g., surfactants) to promote pore formation. Thus, their limited amount of mesopores probably resulted only from interparticle porosity. Interestingly, silanization affected each sample differently in terms of surface area. Although introducing organic groups from TMSCl decreased the SSA_BET_ of all samples, the synthetic nanoparticles retained most of the SSA. Hence, commercial silica (CA) SSA_BET_ dropped dramatically (from 526 to 278 m^2^ g^−1^). In contrast, surface area reduction was reasonably contained for RH and SG (17.6% and 28.4%) compared to the initial SSA_BET_ value. It is worth noting that AC wider pore size and significant surface accessibility might have allowed a higher amount of silyl groups to be anchored on the commercial silica, hindering nitrogen physisorption.

**Figure 4:**
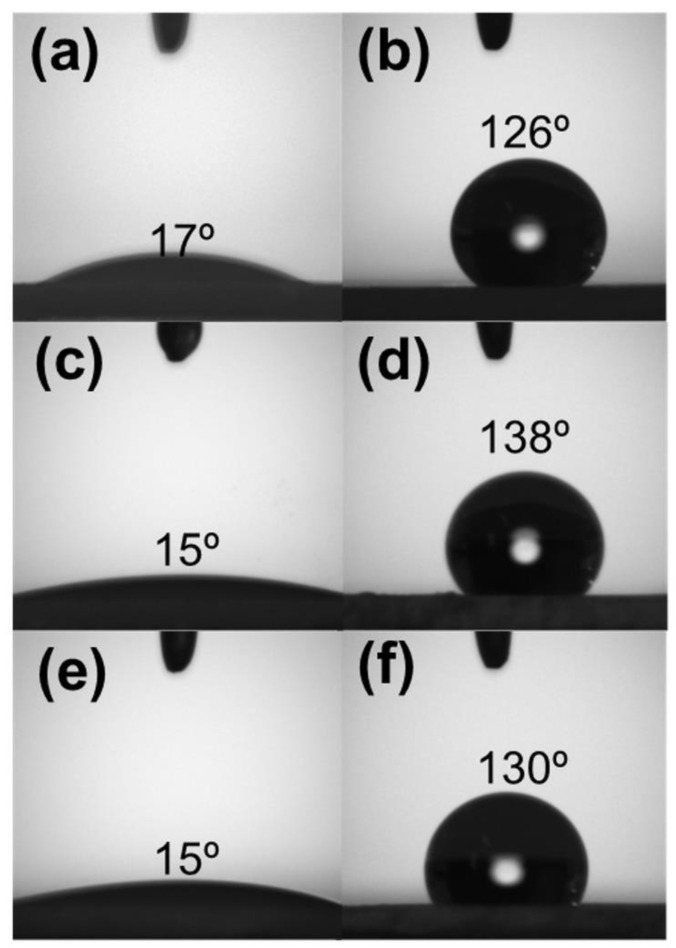
The contact angle for unmodified (left) and modified (right) SiO_2_ surface: (**a**,**b**) AC, (**c**,**d**) SG, and (**e**,**f**) RH.

Besides influencing the SSA, silanization also altered the silica surface hydrophilic character by replacing Si-OH groups on the SiO_2_ with Si-O-Si-(CH_3_)_3_ moieties. Since Cl^−^ is a good leaving group for nucleophilic substitution reactions, silica surface hydrophobization using TMSCl was strongly favored [33]. Figure 4 shows the contact angle measurements performed on pristine and TMSCl-functionalized silica powders. The surface properties of each silica sample changed dramatically, switching from a very hydrophilic to a remarkably hydrophobic behavior. Such a feature is supposed to improve silica affinity for RhB, enhancing the adsorption rate and promoting the incorporation of silica nanoparticles into the PLA fibers.

**Figure 5:**
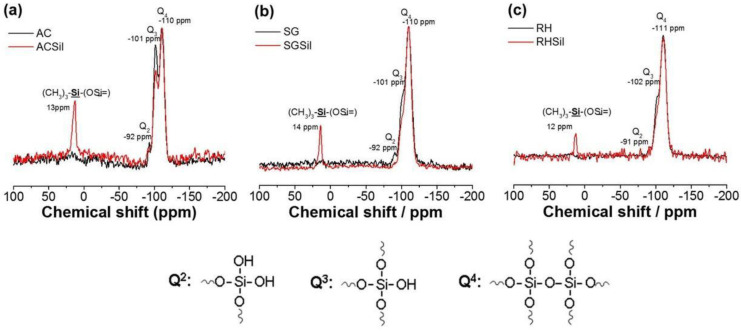
H^1^ RMN for nanoparticles with unmodified and modified SiO_2_ surfaces: (**a**) AC, (**b**) SG, and (**c**) RH.

Since XRD could not detect any structural changes brought about by silanization, ^29^Si NMR spectra were collected on the SiO_2_ surface-modified nanoparticles. In both nanoparticles, we detected the silica surface species referred to as Q^2^–Q^4^ according to the number of siloxane bonds (Figure 5). Specifically, the non-functionalized AC (i.e., pure silica) showed three signals corresponding to chemical shift values of −110, −101, and −92 ppm and representing the Q^2^, Q^3,^ and Q^4^ units, respectively. On the other hand, functionalized silica presented a 13 ppm shift associated with the Si-O-Si-(CH_3_)_3_ unit, aside from the pure silica signals. In addition, an intensity decrease of the signals corresponding to Q^2^ and Q^3^ was observed in the silanized silica.

**Figure 6:**
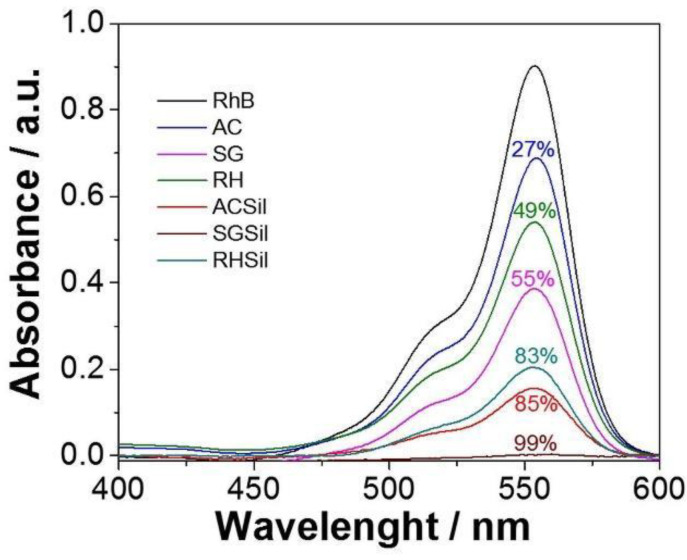
RhB dye adsorption by nanoparticles with unmodified and modified SiO_2_ surfaces after 24 h.

After being characterized, all silica nanoparticles were tested for RhB adsorption. Figure 6 illustrates the UV-Vis absorption spectra of RhB solutions (5 ppm) depending on different adsorbents used for the dye removal. In addition, optical absorbance (i.e., color) attenuation was calculated from the 550 nm absorption peak of the initial RhB solution (blank).

The TMSCl-functionalized silica performed better than their unmodified counterparts in terms of RhB removal, confirming the crucial role played by surface hydrophobization. In particular, SGSil proved to be the most effective adsorbent, achieving a 99% color removal rate. The RHSil reached a dye removal rate (83%) similar to the CS one (85%). Not surprisingly, adsorption results for the silanized samples were perfectly in line with the SSA data (SGSil > ACSil > RHSil). Despite mesopores being large enough to accommodate the RhB molecule (length 1.8 nm, width 0.7 nm [68]), most adsorption took place on the external surface of RHS, with the dye removal percentage similar to the CS value. In addition, steric hindrance due to rhodamine aromatic groups reasonably hampered dye access to CS mesopores lowering the adsorption rate. Apart from the surface area, other phenomena such as electrostatic, hydrogen bond, and hydrophobic interactions must be considered for RhB adsorption on silica [69,70]. Regarding electrostatic interactions, dye removal can exploit the opposite charges on RhB molecules and SiO_2_ surfaces. The former is a cationic dye, while the latter possesses a negatively charged surface as verified by zeta potential measurements (−35, −32, and −31 mV, for ACSil, SGSil, and RHSil, respectively). Consequently, even the non-functionalized samples demonstrated the ability to adsorb RhB. Nonetheless, electrostatic interactions are insufficient to ensure a high level of adsorption. Therefore, effects brought about by surface silanization, like van der Waals interactions mediated by the TMSCl methyl group, should be considered [69]. Finally, it has already been reported how the existence of hydrogen bonds between the hydroxyl groups on the silica surface and the carboxyl groups on RhB [70,71] may further enhance the adsorbent–adsorbate interaction.

**Figure 7:**
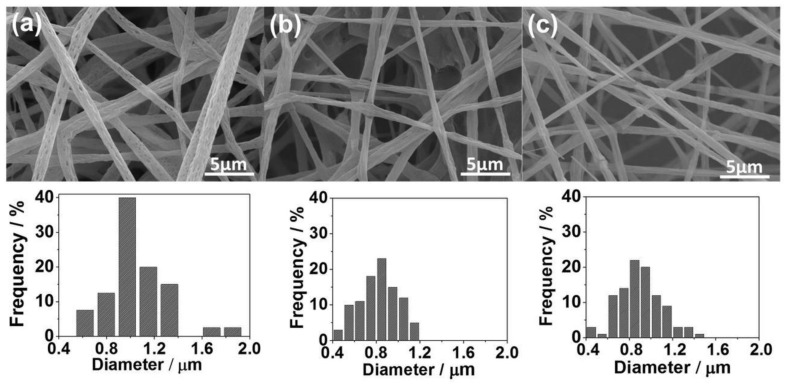
SEM images and fiber diameter histograms of PLA:SiO_2_ membranes: (**a**) pure PLA, (**b**) SGSil, and (**c**) RHSil.

RH and SG SiO_2_-modified nanoparticles showed significant efficiency for RhB dye removal. Thus, both SiO_2_ samples were deposited onto a PLA membrane using the electrospinning method. Figure 7 exhibits the SEM images for the pure PLA fibers and the PLA:SiO_2_ nanocomposites. Initially, it can be seen that the SiO_2_ insertion preserved the fiber shape with a rough appearance and diameter between 0.4 and 1.2 µm. However, regardless of the size distribution of the fiber diameters, it was verified from the histograms that the addition of SiO_2_ nanoparticles promoted a decrease in the size and greater homogeneity of the fibers compared with the pure PLA (0.6–2 µm). This result may have been caused due to the intercalation of the nanoparticles between the PLA polymer chains, causing a decrease in polymer entanglement. Consequently, the electrospinning process favored the stretching of the polymer, resulting in more homogeneous fibers with smaller diameters [72]. Thus, the PLA:SiO_2_ nanocomposite membranes in both cases allowed a gain of morphological properties of the fibers, indicating a good interaction between particle and polymer.

**Figure 8:**
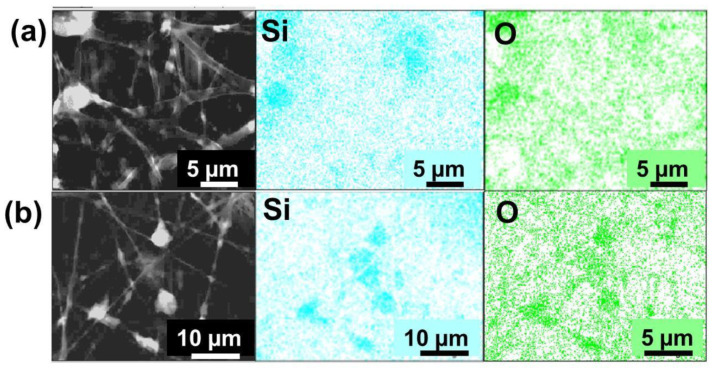
SEM-EDX images of PLA:SiO_2_ membranes: (**a**) SGSil and (**b**) RHSil.

The presence of SiO_2_ nanoparticles and their distribution in the PLA fibers were verified in the composites by energy dispersive X-ray spectroscopy (EDS), as shown in Figure 8. In the fibers of PLA:SiO_2_ SGSil and PLA:SiO_2_ RHSil, the detection of the element silicon (Si) in superposition with oxygen (O) demonstrated the presence of SiO_2_ in the PLA fibers. In addition, the mappings obtained suggest a distribution in the membrane situated in separate regions, which indicates a certain degree of accumulation of the particulates. Such a result may be due to the high SiO_2_ Sil concentration, 40% (w w^−1^), favoring the interaction of particles with each other in the polymer solution. In this sense, both fibers showed a behavior, regardless of the SiO_2_ agglomeration, of being more elucidated for the SiO_2_ extracted from rice husks (Figure 8b).

**Figure 9:**
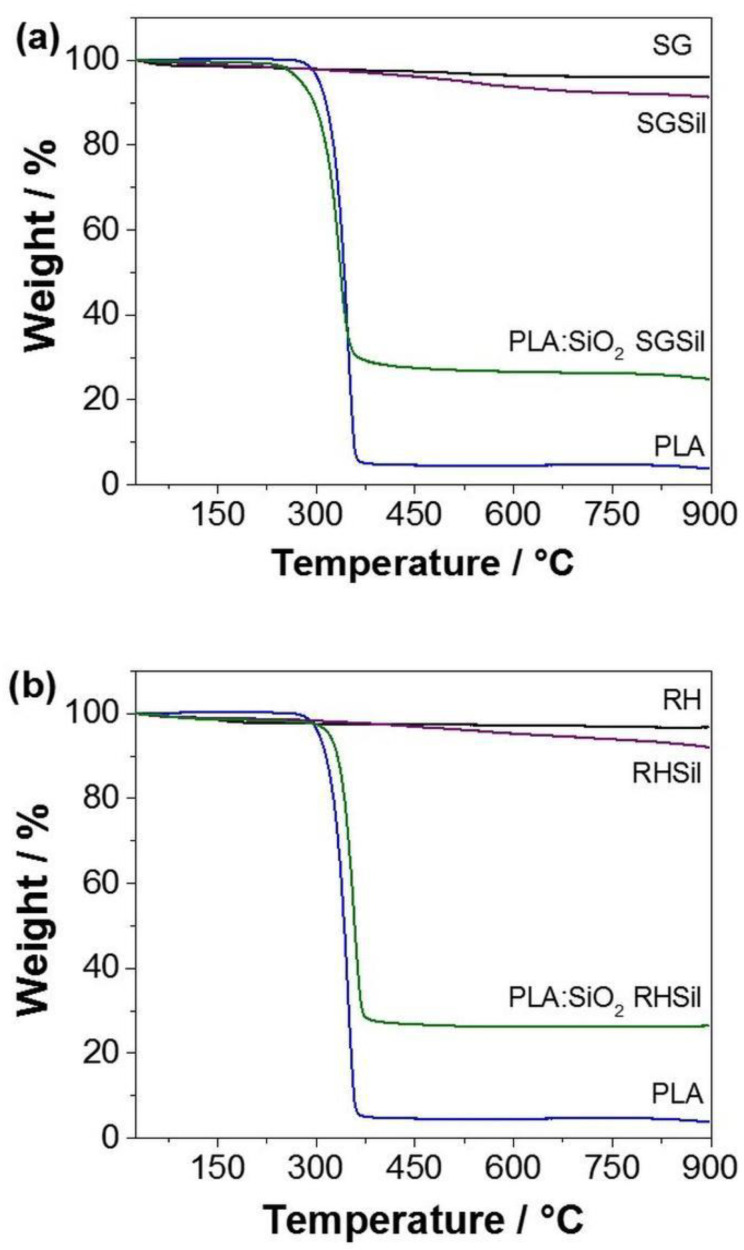
Thermogravimetric analysis of PLA:SiO_2_ membranes: (**a**) SGSil and (**b**) RHSil.

After confirming the presence of the particulate materials in the PLA fibers, thermogravimetric analyses were performed (Figure 8) to quantify the silica mass present in the composites. The determination of particulates in the PLA:SiO_2_ (SGSil and RHSil) fibers was obtained by comparing the PLA and the silica pure nanoparticles’ loss events. The TGA curves show that the non-functionalized samples (SG and RH) had a slight loss in mass due to the adsorbed water loss. On the other hand, in the functionalized samples (SGSil and RHSil), a greater mass loss was observed (9% and 8%, respectively). These values were associated with the additional contribution related to the loss of organic material from surface modification (TMSCl). Concerning pure PLA, a considerable mass loss was observed at 350 °C due to the PLA thermal decomposition [51,55]. The remaining PLA mass probably corresponds to ash formation with increasing temperature. The composite materials of PLA:SiO_2_ SGSil and PLA:SiO_2_ RHSil showed a remaining group of about 27%. This percentage refers to the silica mass present in the fibers. Considering the polymeric solution added 40% (w w^−1^) silica and the functionalized material nanoparticles lost about 9% (Figure 9), the silica mass is expected to be 31%. Thus, the value found in both TGA curves is within the desired values, demonstrating the complete incorporation of the 40% SiO_2_ into the PLA fibers.

**Figure 10:**
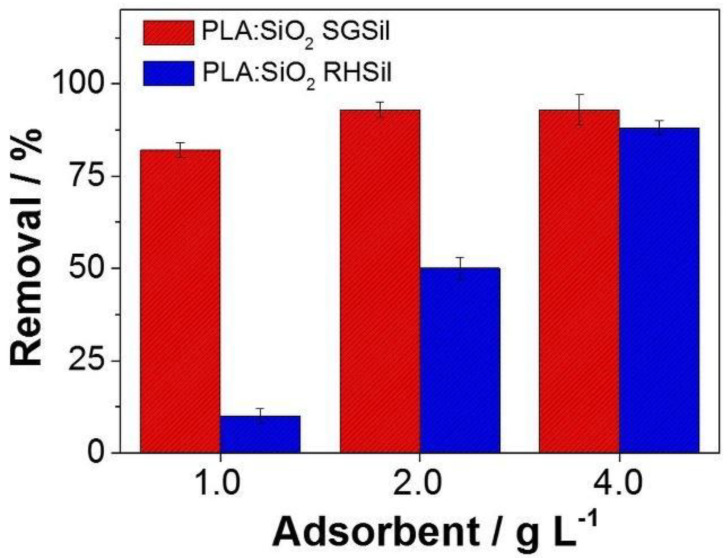
PLA:SiO_2_ membrane adsorption removal against RhB dye.

After characterization, PLA:SiO_2_ SGSil and PLA:SiO_2_ RHSil composite membranes were evaluated for RhB adsorption (5 mg L^−1^). Preliminary experiments were conducted to establish the maximum capacity adsorption, as shown in Figure 10. Initially, PLA:SiO_2_ RHSil fibers were shown to be effective in removing RhB over the entire range of adsorbent concentrations (1–4 g L^−1^). A maximum adsorption capacity of 93% was achieved for 2 g L^−1^. Conversely, the increase in RhB adsorption of PLA:SiO_2_ RHSil was more sensitive to the adsorbent concentration. The highest capacity adsorption (83%) was reached with the maximum concentration evaluated (4 g L^−1^). The difference in RhB dye adsorption by the membranes can be correlated to the characteristics of the SiO_2_-modified nanoparticles. The result observed in Figure 10 corroborates with the free adsorbent particles in Figure 6. In that case, SGSil (99%) showed superior adsorption compared to RHSil (83%). This behavior is attributed to the RHSil’s lower surface area (202 m^2^ g^−1^) after surface modification (Table 1). Thus, as expected, PLA:SiO_2_ RHSil showed less effectiveness compared to PLA:SiO_2_ SGSil in similar conditions. Moreover, silica immobilized in polymer fibers can reduce the number of SiO_2_ active sites available for dye adsorption. Additionally, the distinct interaction of nanoparticles with the polymeric matrix influences the distribution of particles on the membrane surface, as demonstrated in Figure 8. Consequently, the surface area decreases, affecting RHSil adsorption performance in the filter membrane.

**Figure 11:**
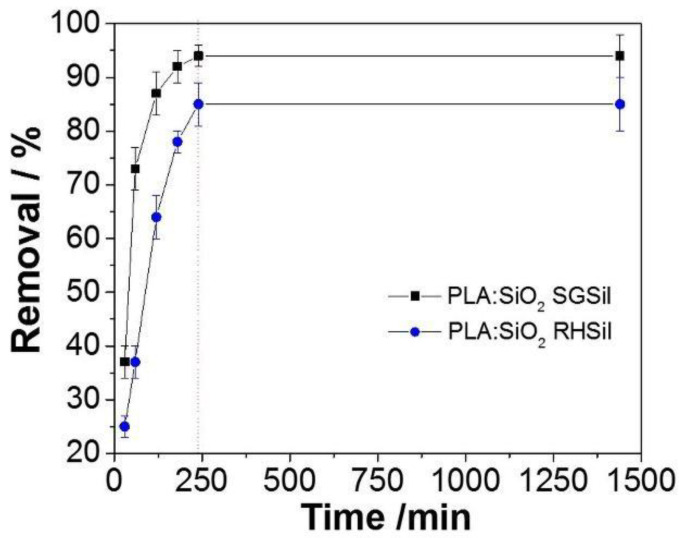
Kinetic study for RhB adsorption using PLA:SiO_2_ nanocomposites.

RhB adsorption kinetic curves are reported in Figure 11. In the observed results, the dye removal percentage increased steadily to 93% by 180 min for PLA:SiO_2_ SGSil. On the other hand, the maximum capacity was slowly reached for PLA:SiO_2_ RHSil up to 240 min. The longer time required for the PLA:SiO_2_ RHSil membranes to achieve equilibrium may be related to its smaller surface area of nanoparticles and agglomeration effect in the fibers, resulting in fewer sites for adsorption. Thus, RhB dye removal for PLA:SiO_2_ RHSil likely needs more diffusion through the fiber membranes resulting in a more extended time exposition for adsorption.

**Figure 12:**
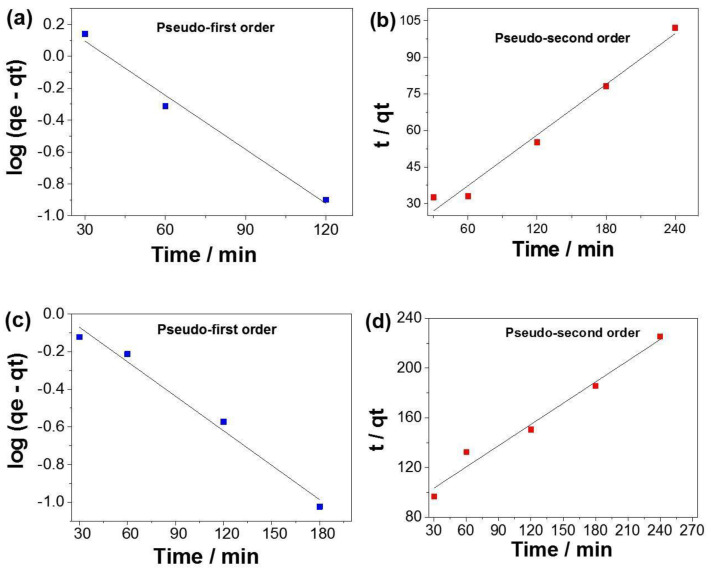
Kinetic assays of RhB dye adsorption (**a**,**b**) PLA:SiO_2_ SGSil and (**c**,**d**) PLA:SiO_2_ RHSil.

The kinetic parameters were determined from linear regression (Figure 12) and are presented in Table 2. According to the correlation coefficient (R^2^) analyses, the pseudo-first-order model best fits the experimental data, showing R^2^ values of 0.9865 and 0.9842 for PLA:SiO_2_ SGSil and PLA:SiO_2_ RHSil, respectively. Based on this model, the calculated *q_e_* values (2.7158 and 1.2956 mg g^−1^) were close to the experimental *q_e_* values (2.3012 and 1.0648 mg g^−1^) established for dye adsorption using PLA:SiO_2_ SGSil and PLA:SiO_2_ RHSil, respectively. This result resembles those in the literature [73,74], which employed SiO_2_-supported nanocomposites as adsorbents for RhB removal. In addition, PLA:SiO_2_ SGSil fibers showed a speed constant (*k*_1_) that was almost twice (0.02602 min^−1^) as high as that of PLA:SiO_2_ RHSil (0.01404 min^−1^), confirming the more difficult diffusion access to adsorption sites.

**Figure 13:**
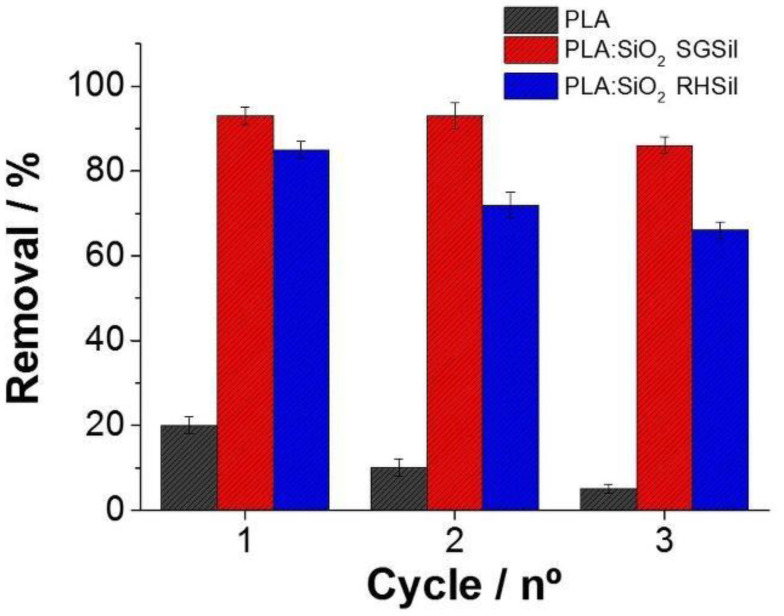
Adsorption cycles for PLA:SiO_2_ nanocomposite fibers.

RhB adsorption/desorption cycles were executed on PLA:SiO_2_ SGSil and PLA:SiO_2_ RHSil to evaluate nanocomposite regeneration/reuse capabilities. Figure 13 shows the RhB dye adsorption during three reuse cycles. PLA pure fibers as an adsorbent achieved a less than 20% removal rate in the 1° cycle. On the other hand, both filter membranes showed adsorptions greater than 80% (1° cycle). Hence, RhB removal was nearly entirely due to the SiO_2_ nanoparticles supported on PLA fibers. In the reuse cycles, all materials demonstrated a decreased capacity for adsorption. In this way, both nanocomposites (PLA:SiO_2_ SGSil and PLA:SiO_2_ RHSil) maintained affinity interactions with RhB dye after desorption, promoting adsorption saturation. As a result, PLA:SiO_2_ SGSil and PLA:SiO_2_ RHSil had decreased adsorption capacities from 93% to 86% and 83% to 68%, respectively, after three reuse cycles.

The current results are consistent with similar works involving dye adsorption on silica-polymer composite fibers. Wu et al. [75] investigated RhB dye adsorption on SiO_2_ nanoparticles supported on polystyrene electrospun fibers. In this study, a 33% maximum dye adsorption was reported. Xu et al. [47] evaluated PAA:SiO_2_ composite nanofiber membranes in the removal of malachite green dye. As a result, the membrane reached 98% dye removal after 240 min in the 1° cycle, maintaining about 70% dye removal after three adsorption/desorption cycles. Therefore, the system absorbent based on PLA:SiO_2_ with TMSCl surface modification showed significant results in adsorption removal. Further, the present adsorptive membrane exhibited comparable results to the “low-cost/low-end” rice husk-based nanocomposite for wastewater treatment.

## 4. Conclusions

Mesoporous SiO_2_ nanoparticles with diameters less than 20 nm, with high purity, and a surface area of 245 m^2^ g^−1^ were obtained by extraction from rice husks (RH). The surface modification of SiO_2_ nanoparticles with TMCS contributed to an increase in RhB dye adsorption from 49% to 83%. This result was comparable with that found for commercial and sol–gel synthesized silica. Furthermore, the modified SiO_2_ nanoparticles [40% (w w^−1^)] were impregnated efficiently into PLA fibers using the electrospinning method. The PLA:SiO_2_ nanocomposite membranes from rice husk showed more than 80% dye removal, maintaining 60% activity after three cycles. In this context, this paper offers a system with high RhB affinity, proving that the silica nanoparticles supported on PLA nanofibers promote easy removal and reuse cycles without significantly impairing adsorption. Silica (e.g., quartz) surface functionalization is already exploited on an industrial scale in direct and reverse minerals flotation. Furthermore, TMSCl is used mainly in organic synthesis as a trimethylsilyl group or anhydrous chloride source. The production fibers have been quickly improved to minimize economic costs for large-scale applications by solution blowing spinning from electrospinning knowledge. Finally, the development of adsorptive membranes from agri-wastes such as rice husks can minimize costs and enable their use in real applications involving the adsorption of organic pollutants in water to favor environmental remediation.

## Figures and Tables

**Table 1:** N_2_ adsorption/desorption results for the SiO_2_ samples before and after silanization.

	Particle Diameter/nm	SSA_BET_ /m^2^ g^−1^	A_Ext_ /m^2^ g^−1^	A_Pore_ /m^2^ g^−1^	D_pore_ /nm
SiO_2_					
AC	16	526.1373	249.6770	276.4603	3.26773
ACSil	16	278.2454	175.4967	102.7487	4.18450
SG	5	536.6674	527.2922	9.3751	4.22250
SGSil	5	384.5106	-	-	4.01733
RH	14	245.8613	225.6661	20.1952	5.12742
RHSil	14	202.5582	-	-	4.83049

**Table 2:** Kinetic parameters of RhB dye adsorption from pseudo-first-order and pseudo-second-order models.

Adsorbent	Experimental	Pseudo-First Order			Pseudo-Second Order		
PLA:SiO_2_	*q_e_* mg g^−1^	*q_e_* mg g^−1^	*k*_1_ min^−1^	R^2^	*q_e_* mg g^−1^	*k*_2_ g mg^−1^ min^−1^	R^2^
SGSil	2.3012	2.7158	0.02602	0.9865	2.8893	7.20 × 10^−3^	0.9817
RHSil	1.0648	1.2956	0.01404	0.9842	1.758	3.74 × 10^−3^	0.9775

## Data Availability

Not applicable.
